# Ultrasound-guided cooled radiofrequency ablation for sacroiliac joint pain in a patient with PsAPASH syndrome: A case report

**DOI:** 10.1016/j.inpm.2026.100742

**Published:** 2026-02-17

**Authors:** Catarina Henriques Afonso, Diogo Ribeiro Martins, Joana Cabete, Inês Camarinha

**Affiliations:** a Department of Physical Medicine and Rehabilitation, Unidade Local de Saúde de S. José, Portugal; b Centro Multidisciplinar da Dor, Unidade Local de Saúde Almada–Seixal, Portugal; c Department of Dermatology, Unidade Local de Saúde de S. José, Portugal; d Member of the European Hidradenitis Suppurativa Foundation, Portugal

**Keywords:** Cooled radiofrequency ablation, Sacroiliac joint, PsAPASH syndrome, Hidradenitis suppurativa, Ultrasound guidance, Interventional pain medicine

## Abstract

PsAPASH syndrome is a rare autoinflammatory disorder comprising psoriatic arthritis, pyoderma gangrenosum, acne, and hidradenitis suppurativa, characterized by severe cutaneous and musculoskeletal manifestations that are often refractory to systemic therapies. Sacroiliitis is a recognized feature of PsAPASH, contributing significantly to functional disability. While cooled radiofrequency ablation has emerged as an effective treatment for chronic sacroiliac joint pain, performing interventional procedures in patients with extensive active hidradenitis suppurativa poses unique challenges due to infection risk from cutaneous lesions near needle insertion sites. We report the first case of ultrasound-guided cooled radiofrequency ablation for sacroiliac joint pain in a patient with PsAPASH syndrome and extensive active hidradenitis suppurativa.

A 22-year-old female with PsAPASH syndrome presented with bilateral sacroiliitis confirmed by MRI and positive provocative maneuvers, refractory to systemic biologic therapy, NSAIDs, and physiotherapy. Diagnostic intra-articular corticosteroid injections provided greater than 50% pain relief, establishing indication for definitive treatment. Due to extensive active hidradenitis suppurativa lesions near planned needle insertion sites, a multidisciplinary approach was employed involving Physical and Rehabilitation Medicine, Dermatology, and Interventional Pain specialists. Ultrasound-guided cooled radiofrequency ablation targeting the L5 dorsal ramus and S1-S2-S3 lateral branches was successfully performed with real-time mapping to avoid affected skin areas, supplemented by prophylactic antibiotic coverage. The procedure was uneventful, with no infectious or other complications. At 9-month follow-up, the patient maintained excellent pain control (mean NPRS 0, peak NPRS 2) with restoration of functional capacity.

This case demonstrates that ultrasound-guided cooled radiofrequency ablation can be safely and effectively performed for sacroiliac joint pain in patients with PsAPASH syndrome, even in the presence of extensive active cutaneous disease, when appropriate multidisciplinary coordination and infection prevention strategies are employed.

## 1 Introduction

PsAPASH syndrome—comprising psoriatic arthritis, pyoderma gangrenosum, acne, and hidradenitis suppurativa (HS)—is a rare autoinflammatory disorder first described in 2015 [1]. It belongs to a spectrum of pyoderma gangrenosum–associated autoinflammatory syndromes that share dysregulation of innate immunity, particularly involving abnormal IL-1, IL-17, and TNF-α signaling [1,2]. The syndrome frequently presents with severe, treatment-resistant cutaneous and musculoskeletal manifestations, posing significant therapeutic challenges [3].

Musculoskeletal involvement, particularly axial spondyloarthritis with sacroiliitis, is a recognized feature of PsAPASH and related syndromes, contributing substantially to functional disability [4]. When sacroiliac joint (SIJ) pain remains refractory to conservative management and systemic immunosuppression, interventional approaches may be considered. Cooled radiofrequency ablation (cRFA) has emerged as an effective minimally invasive treatment for chronic SIJ pain, producing larger and more consistent neural lesions than conventional radiofrequency techniques [5,6].

While fluoroscopy is the standard imaging modality for SIJ procedures, ultrasound guidance offers real-time soft tissue visualization and radiation-free imaging [7]. However, evidence for ultrasound-guided cRFA remains limited. Furthermore, performing interventional procedures in patients with HS poses unique challenges due to the potential proximity or overlap of active cutaneous lesions with needle insertion sites, as well as the risk of infection when these lesions are infected.

To our knowledge, this is the first reported case of ultrasound-guided cooled radiofrequency ablation for sacroiliac joint pain in a patient with PsAPASH syndrome. This case demonstrates the feasibility, safety, and efficacy of this approach in the challenging context of extensive active cutaneous disease.

## 2 Case report

**Fig. 1:**
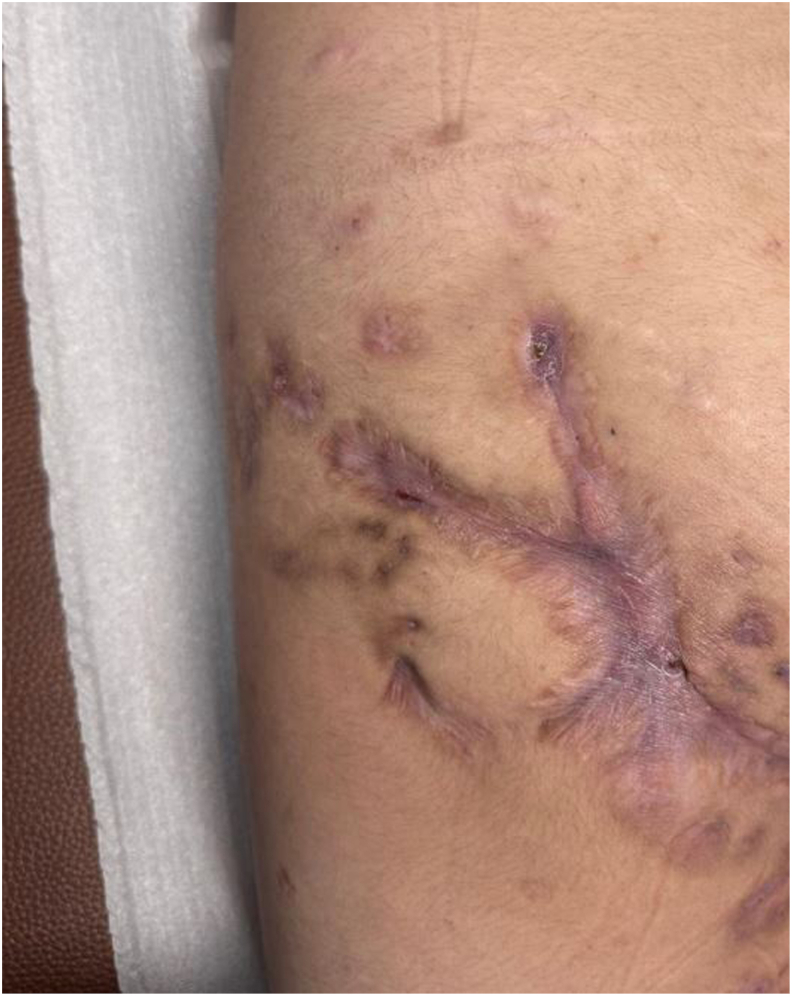
Clinical photographs showing active hidradenitis suppurativa lesions in the gluteal region with nodules and fistulous tracts.

A 22-year-old female with an unremarkable family history and no known drug allergies presented with a complex clinical course of hidradenitis suppurativa (HS) dating back to age 10. The condition was characterized by recurrent painful nodules, abscesses, and tunnels involving the perineal and inguinal regions (Fig. 1). Over subsequent years, she developed pyoderma gangrenosum, psoriasis, acne conglobata, and psoriatic arthritis, consistent with the phenotype of PsAPASH syndrome.

Genetic testing revealed heterozygosity for the NOD2 gene. Flow cytometric analysis demonstrated an increased Th17 and Th17/Th1 profile with expansion of central memory CD8^+^ T cells, supporting dysregulation of both innate and adaptive immune pathways and a predominant IL-17–driven inflammatory response.

The patient underwent multiple therapies, including adalimumab, infliximab, secukinumab, anakinra, ustekinumab, and adjuvant methotrexate, without achieving sustained remission. She eventually attained partial disease control with combined therapy consisting of brodalumab, though weekly, and upadacitinib 30 mg daily, resulting in improvement of both cutaneous and articular manifestations.

Since age 19, the patient has experienced recurrent episodes of inflammatory back pain with morning stiffness, predominantly affecting the lumbar spine and bilateral sacroiliac joints, and to a lesser extent, the shoulders, wrists, and cervical spine. Symptoms were only partially controlled with oral nonsteroidal anti-inflammatory drugs (NSAIDs).

At age 20, she reported progressive worsening of bilateral gluteal and sacroiliac pain, inflammatory in nature, refractory to analgesics, and associated with significant functional limitation. Physical examination revealed localized tenderness over the right SIJ. Provocative maneuvers were performed, with distraction test and sacral thrust reproducing the patient's characteristic pain, supporting the diagnosis of SIJ dysfunction.

**Fig. 2:**
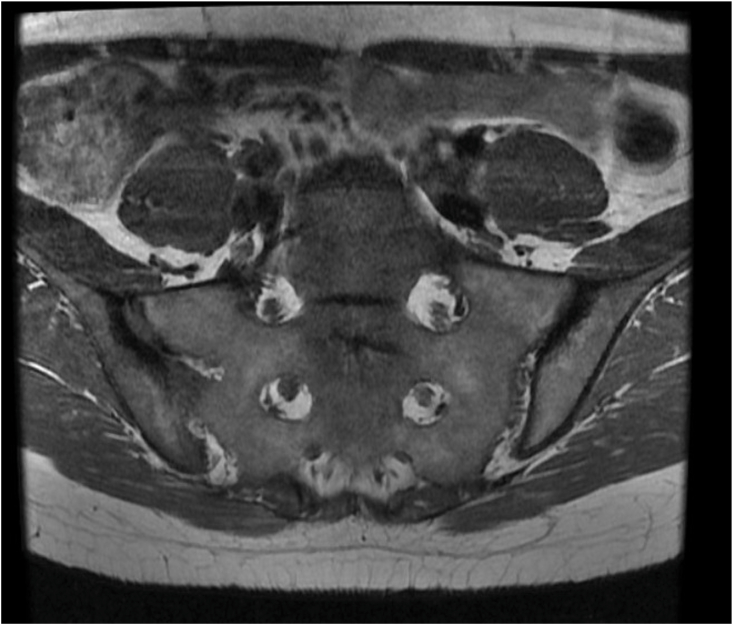
Pelvic MRI findings: T1-weighted coronal image showing bilateral subchondral sclerosis of the sacroiliac joints.

Magnetic resonance imaging (MRI) of the pelvis demonstrated bilateral symmetric subchondral sclerosis involving the synovial portions of both sacroiliac joints, accompanied by bone marrow edema, capsulitis, and micro- and macroerosions, consistent with active bilateral sacroiliitis (Fig. 2). Additionally, mild erosive changes at the pubic symphysis and inflammatory subcutaneous foci in the gluteal region were noted, the latter compatible with active HS.

Given these findings, the patient was referred to the Physical and Rehabilitation Medicine (PRM) department for multidisciplinary management. The PRM team prescribed physiotherapy—ideally hydrotherapy—but due to extensive cutaneous lesions, only land-based rehabilitation was feasible. After four months of therapy, no significant improvement was observed.

**Fig. 3:**
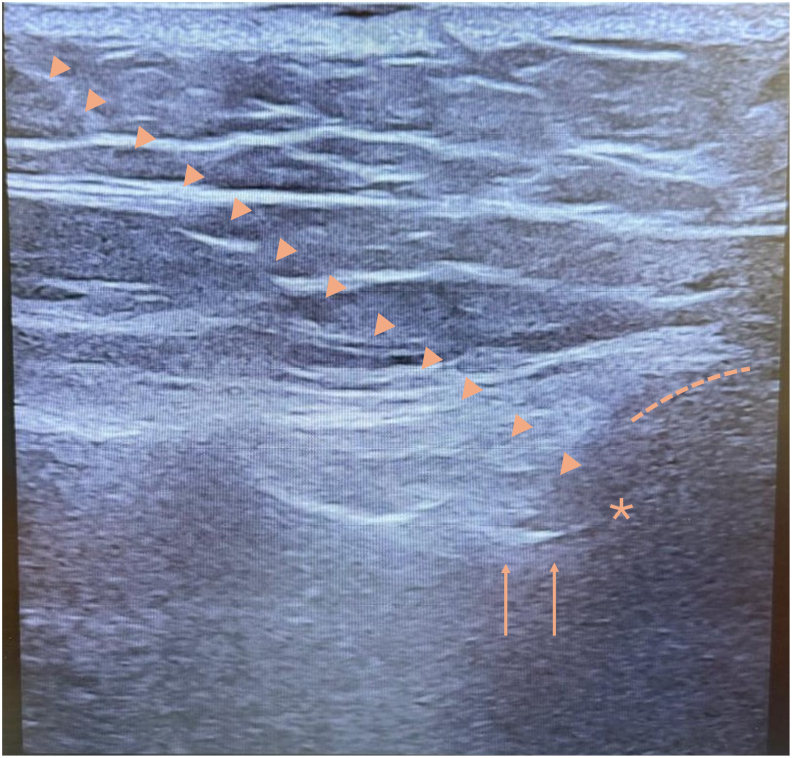
Ultrasound image demonstrating needle positioning for intra-articular sacroiliac joint injection (arrowheads), obtained with the transducer oriented in an oblique transverse plane over the posterior sacroiliac joint. The joint space is indicated by an asterisk. The dotted lines outline the bony surface of the ilium and the arrows point at the dorsal surface of the sacrum.

Given the persistence of disabling pain and limited response to pharmacologic and rehabilitative interventions, interventional management was pursued. Under ultrasound guidance, a combined intra-articular and peri-articular corticosteroid injection was performed in the right SIJ using 4 mL total volume containing 2% lidocaine and 14 mg betamethasone. Approximately 2.0-2.5 mL was delivered intra-articularly under direct visualization, followed by intentional peri-articular infiltration of the remaining volume into the posterior ligamentous complex to target both the synovial joint and the richly innervated capsular-ligamentous structures that contribute to SIJ pain. This resulted in approximately 60% pain reduction and improved quality of life; this effect was sustained over time. One month later, a similar injection was administered to the left SIJ (Fig. 3), yielding complete pain relief for two weeks followed by partial symptom recurrence. This response—exceeding the 50% pain reduction threshold—provided diagnostic confirmation and established an indication for radiofrequency ablation (RFA).

**Fig. 4:**
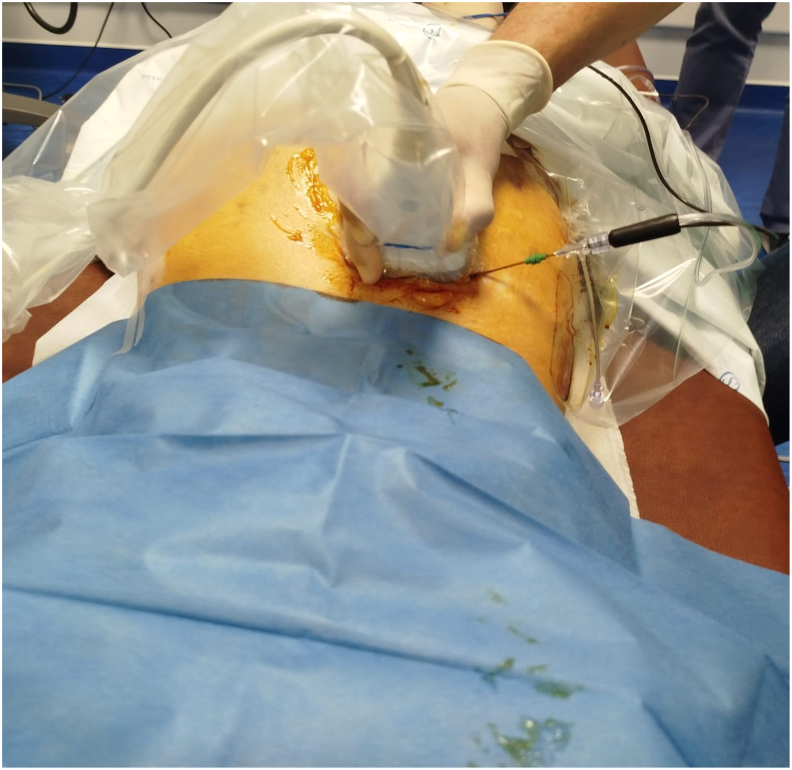
Ultrasound-guided cooled radiofrequency ablation procedure showing probe placement.

In light of this partial but clinically meaningful response, cRFA targeting the lateral branches of S1-S2-S3 and the dorsal ramus of L5 was proposed on the left SIJ for longer-lasting analgesia. However, the proximity of active HS lesions raised concerns regarding procedural infection risk. Since the classic RFA protocol for SIJ is performed under fluoroscopy, which would not properly warrant avoidance of the lesions, the case was therefore discussed with the Dermatology department, and a coordinated plan was established, including antibiotic flare prophylaxis with clindamycin 300 mg bid and real-time ultrasound mapping to avoid affected skin areas. Under local anesthesia with 1% lidocaine (10 mL), cRFA was performed using the Coolief® system (Avanos Medical, Alpharetta, GA, USA) targeting the sacral lateral plexus—a complex neural network derived from the L5 dorsal ramus and S1-S2-S3 lateral branches (Fig. 4).

Procedural technique: With the patient in prone position, ultrasound guidance was used to perform a systematic denervation approach. The S1, S2, and S3 sacral foramina were first identified using longitudinal and transverse ultrasound planes. For each foramen, the initial cannula was positioned immediately lateral to the foramen under real-time visualization, corresponding to the 3 o'clock position. Subsequently, by maintaining the foramen visualized on the medial aspect of the ultrasound probe and tilting the probe diagonally approximately 10-20° cranially and caudally, additional cannula positions were achieved corresponding to the 2 and 4 o'clock orientations relative to each foramen. This dynamic probe angulation technique allowed precise control of the cannula-foramen distance while ensuring comprehensive coverage of the sacral lateral branches, analogous to the fluoroscopy-guided approach. Three cRFA lesions were performed at the 2, 3, and 4 o'clock positions lateral to each of the S1, S2, and S3 foramina, with an additional lesion targeting the L5 dorsal ramus at the junction between the sacral ala and the base of the superior articular process of S1, for a total of 10 cRFA lesions, followed by the injection of a total of 8 mg of dexamethasone distributed across the 10 lesions. Before performing the lesions, sensory and motor stimulation were administered to confirm correct cannula placement and to avoid potential unintended lesions – sensory stimulation at 50 Hz was positive at 0.2-0.5 V and motor stimulation at 2Hz was negative up to 2 V.

The procedure was uneventful, and the patient was discharged pain-free with oral clindamycin 300 mg twice daily for seven days.

The patient was evaluated at one, three, six and nine-months follow-up, maintaining excellent pain control (mean NPRS 0, peak NPRS 2), reporting restoration of full functional capacity and marked improvement in quality of life.

## 3 Discussion

Written informed consent was obtained from the patient for publication of this case report and accompanying clinical images.

This case illustrates several important clinical challenges in the management of PsAPASH syndrome: the complexity of achieving disease control with systemic therapies, the significant burden of musculoskeletal involvement, and the need for innovative, multidisciplinary approaches to address refractory symptoms in the presence of active cutaneous disease.

### 3.1 Systemic disease management

The pathogenesis of PsAPASH involves dysregulated IL-1β and IL-17 signaling pathways, leading to chronic systemic inflammation with both cutaneous and musculoskeletal manifestations [4]. Our patient's flow cytometric analysis demonstrated increased Th17 and Th17/Th1 cell populations with expansion of central memory CD8^+^ T cells, providing objective immunological evidence supporting the diagnosis of PsAPASH and confirming the autoinflammatory nature of her disease.

Despite sequential trials of multiple biologic agents targeting different inflammatory pathways (TNF-α inhibitors: adalimumab, infliximab; IL-17 inhibitors: secukinumab; IL-17 receptor blocker: brodalumab; IL-12/23 inhibitor: ustekinumab; IL-1 antagonist: anakinra; JAK inhibitor: upadacitinib), our patient achieved only partial control even under combination therapy. This therapeutic resistance is characteristic of syndromic HS and underscores the need for multidisciplinary management strategies that extend beyond systemic immunomodulation [3].

### 3.2 Sacroiliac joint involvement and interventional management

Musculoskeletal manifestations, particularly axial involvement with sacroiliitis, are well-recognized features of PsAPASH and related syndromes such as PASS *(Pyoderma gangrenosum, Acne, Hidradenitis suppurativa, and Spondyloarthritis)* [4]. Our patient's bilateral sacroiliitis—confirmed by MRI findings of subchondral sclerosis, bone marrow edema, and erosions—contributed significantly to functional disability despite systemic biologic and small-molecule therapy. Following diagnostic intra-articular corticosteroid injections, the patient achieved approximately 60% pain reduction in the right SIJ and complete (though transient) relief in the left SIJ. This response exceeded the recommended 50% threshold for proceeding to radiofrequency ablation [8], confirming the SIJ as the primary pain generator and establishing a clear indication for definitive denervation treatment.

Cooled radiofrequency ablation (cRFA) has emerged as a superior alternative to conventional RFA for SIJ denervation, producing larger, more consistent lesions through active probe cooling [5]. The procedure targets the lateral branches of S1–S3 and the dorsal ramus of L5, which constitute the primary posterior innervation of the SIJ [6]. Studies report that most cRFA-related adverse events are mild and transient, with no serious complications or nerve injuries [5,7].

### 3.3 Ultrasound guidance in the context of active cutaneous disease

While fluoroscopy remains the traditional imaging modality for SIJ procedures, ultrasound guidance offers several theoretical advantages, including real-time visualization of soft tissues, absence of ionizing radiation, and the ability to avoid anatomical structures or pathological areas [7]. In our patient, the presence of extensive, active HS lesions in close proximity to the planned needle insertion sites posed a significant risk of procedural infection and precluded standard fluoroscopic approaches.

Ultrasound guidance enabled real-time mapping of cutaneous involvement, allowing for dynamic selection of safe needle trajectories that avoided compromised skin. This approach was supplemented by prophylactic antibiotic coverage and close collaboration with the Dermatology department to optimize procedural timing relative to disease activity. To our knowledge, this is the first report describing the use of ultrasound-guided cRFA in the context of active HS or any autoinflammatory syndrome affecting the skin.

The main limitation of ultrasound guidance lies in its reduced precision for deep or anatomically variable structures and its operator dependence [7]. However, cadaveric and clinical studies have demonstrated that ultrasound-guided needle placement along the lateral sacral crest can provide satisfactory visualization of bony landmarks and reproducible access to target nerves [7]. In selected patients - such as those with contraindications to fluoroscopy, active cutaneous lesions, or specific anatomical considerations - ultrasound guidance may represent a valuable alternative, provided that operators have adequate training and experience.

### 3.4 Infection risk management

The decision to proceed with an invasive procedure in the setting of active HS required careful risk-benefit assessment. HS lesions may be colonized by polymicrobial flora and pose a theoretical risk of introducing infection into deeper structures. Our multidisciplinary approach included: (1) careful procedural planning with Dermatology input, (2) real-time ultrasound mapping to avoid affected skin, (3) meticulous aseptic technique, and (4) prophylactic antibiotic coverage with clindamycin - an agent with good penetration into skin and soft tissue and activity against common HS pathogens. The absence of infectious or other complications supports the feasibility of this approach when appropriately planned.

### 3.5 Clinical outcomes and implications

While our patient achieved excellent middle-term outcomes with complete pain relief at nine months, the long-term durability of cooled radiofrequency ablation for sacroiliac joint pain remains an important consideration. Studies of cRFA for SIJ pain report initial pain relief durations of approximately 5.5 months, with some patients experiencing sustained benefit extending to two years post-procedure. Notably, repeat cRFA procedures have been shown to provide even longer pain relief (9.0 months) compared to initial treatments [9].

In the context of PsAPASH syndrome, where ongoing systemic inflammation may contribute to continued joint damage despite biologic therapy, the durability of denervation procedures may be further compromised. The natural history of the underlying autoinflammatory process - with its propensity for relapsing and remitting disease activity - suggests that our patient may eventually require repeat cRFA. Repeat procedures are both feasible and effective, with evidence demonstrating increased duration of benefit and substantial reductions in healthcare utilization costs [6]. Nonetheless, even relief lasting 6-12 months represents a clinically meaningful improvement in quality of life and functional capacity, particularly when integrated into a comprehensive treatment strategy that includes systemic immunomodulation, physiotherapy, and ongoing multidisciplinary care.

In cases where repeated cRFA yields diminishing returns or fails to provide adequate pain control after at least six months of conservative and interventional management, minimally invasive sacroiliac joint fusion may be considered as a definitive treatment option, with studies demonstrating substantial clinical benefit in over 90% of carefully selected patients [10]. However, in the setting of active cutaneous disease characteristic of PsAPASH, surgical fusion would require careful multidisciplinary planning to minimize infection risk, similar to the coordinated approach used for our patient's cRFA procedure. Long-term follow-up studies are needed to determine the optimal timing for repeat procedures in patients with inflammatory spondyloarthropathies and to identify predictors of treatment durability in this unique patient population.

This is a single case report with short-term follow-up, and longer observation is needed to assess durability of effect. The relative contributions of ultrasound guidance versus the cRFA technique itself to the successful outcome cannot be definitively determined. Further studies comparing ultrasound-guided and fluoroscopy-guided cRFA in diverse patient populations are warranted.

This case demonstrates that ultrasound-guided cooled radiofrequency ablation can be safely and effectively performed for sacroiliac joint pain in patients with PsAPASH syndrome, even in the presence of extensive active hidradenitis suppurativa. Success depends on careful multidisciplinary coordination among Physical and Rehabilitation Medicine, Dermatology, and Interventional Pain specialists, with strategic use of ultrasound guidance to avoid affected skin areas and appropriate antibiotic prophylaxis to minimize infection risk. This approach offers a valuable option for managing refractory musculoskeletal pain in complex autoinflammatory syndromes where standard interventional techniques may be contraindicated. Our experience suggests that interventional pain management should be considered as part of comprehensive care for these challenging patients, and that further research is needed to establish standardized protocols and evaluate long-term outcomes in this population.
